# Effects of ambient noise on detectability and localization of avian songs and tones by observers in grasslands

**DOI:** 10.1002/ece3.1847

**Published:** 2015-12-29

**Authors:** Nicola Koper, Lionel Leston, Tyne M. Baker, Claire Curry, Patricia Rosa

**Affiliations:** ^1^ Natural Resources InstituteUniversity of Manitoba70 Dysart RoadWinnipegManitobaR3T 2M7Canada; ^2^ Department of Biological SciencesUniversity of AlbertaCW 405Biological Sciences BuildingEdmontonAlbertaT6G 2E9Canada; ^3^ TERA Environmental Consultants815 8 Ave SWCalgaryAlbertaT2M 2M8Canada

**Keywords:** Distance estimation, energy development, grasslands, perceptibility

## Abstract

Probability of detection and accuracy of distance estimates in aural avian surveys may be affected by the presence of anthropogenic noise, and this may lead to inaccurate evaluations of the effects of noisy infrastructure on wildlife. We used arrays of speakers broadcasting recordings of grassland bird songs and pure tones to assess the probability of detection, and localization accuracy, by observers at sites with and without noisy oil and gas infrastructure in south‐central Alberta from 2012 to 2014. Probability of detection varied with species and with speaker distance from transect line, but there were few effects of noisy infrastructure. Accuracy of distance estimates for songs and tones decreased as distance to observer increased, and distance estimation error was higher for tones at sites with infrastructure noise. Our results suggest that quiet to moderately loud anthropogenic noise may not mask detection of bird songs; however, errors in distance estimates during aural surveys may lead to inaccurate estimates of avian densities calculated using distance sampling. We recommend caution when applying distance sampling if most birds are unseen, and where ambient noise varies among treatments.

## Introduction

It has long been recognized that detectability of birds during field surveys is imperfect (e.g., Anderson 2001; Johnson 2008; Efford and Dawson 2009). Naïve (unadjusted) surveys may produce biased measures of bird abundance, because numbers of birds detected is a function of both abundance and the probability of their detection, which may vary among study sites (Anderson 2001). Conversely, statistical methods that adjust for detection probability may create biases greater than those of unaltered indices (Efford and Dawson 2009); however, the effect and risk of these biases varies among habitat types (Johnson 2008). It is, therefore, important to evaluate detectability of birds under a variety of conditions that are likely to be encountered during field surveys, to understand potential biases and so that costs and benefits of adjusting for imperfect detectability can be assessed.

Background noise in the survey environment is one factor that is likely to affect both detectability (detection of birds that are there) and localization (accuracy in estimating locations of birds detected) of birds (Pacifici et al. 2008; Blickley and Patricelli 2010; Ortega and Francis 2012), but the effect of background noise on detection bias remains poorly understood (Simons et al. 2007). Masking and distraction can skew accurate localization of cues by observers (e.g., field researchers) in the presence of ambient noise. Distraction (or informational masking) refers to decreased detection of or discrimination among signals due to the presence of another irrelevant stimulus (Durlach et al. 2003). This can occur in the presence of ambient sounds at any amplitude or frequency. In contrast, frequency masking refers to the case in which both the signal and background noise occur simultaneously and within similar frequency bands, and as a result the receiver cannot distinguish between the signal and the background sounds (Cooke and Lu 2010). For frequency masking to occur, the amplitude of background noise must be sufficiently high, there must be an overlap between noise and signal frequencies, and the signal‐to‐noise ratio must be low (Dooling and Blumenrath 2013; hearafter, frequency masking is referred to as masking). Many anthropogenic noises, such as traffic, are dominated by lower sound frequencies; therefore, anthropogenic noise may be more likely to mask those bird songs that are also produced at relatively low frequencies (e.g., Hu and Cardoso 2009; Blickley and Patricelli 2010). Additionally, low‐frequency ambient noise may mask high‐frequency signals, but not the inverse (Moore 1994). Therefore, the potential for anthropogenic noise to mask bird signals is high.

Distraction results from different mechanisms than masking. If background noise is cognitively demanding, it diminishes the attentional space available for the simultaneous processing of other cues (North and Hargreaves 1999). While observers differ in their ability to cope with distracting noises, exposure to irrelevant background sound tends to reduce performance in completing relatively complex tasks (Cassidy and MacDonald 2007). While it is perhaps not surprising that loud anthropogenic noises can mask detection of birds by observers (e.g., Simons et al. 2007), even low amplitudes of background noise can distract observers (Smith 1989) and consequently impede an observer's ability to detect or estimate distances to bird calls and songs. Thus, an acoustic stimulus can become a distraction at very low amplitudes, even at levels equivalent to that of ordinary human speech (Banbury et al. 2001).

Oil wells and natural gas compressor stations produce noise at a wide range of frequencies that may mask birdsong or distract observers. Energy infrastructure has been negatively associated with abundance of birds (e.g., Ingelfinger and Anderson 2004; Blickley and Patricelli 2010; Ortega and Francis 2012), in some cases because of the noise that infrastructure produces (e.g., Francis et al. 2012). However, with studies near infrastructure, it can be difficult to disentangle the effects of noise pollution on bird abundance from effects of reduced detection or localization ability by observers (Alldredge et al. 2007; Blickley and Patricelli 2010).

Regardless of the level of ambient noise, detectability declines as distance to individual birds from observers increases; therefore, over the last two decades, distance sampling has been frequently used to adjust abundance estimates of birds detected in the field (e.g. Buckland et al. 1993; Alldredge et al. 2006; Kissling and Garton 2006). However, observers vary in their ability to detect and estimate locations of birds based on acoustic cues (Alldredge et al. 2007), making estimates of detectability and abundance based on specific distribution assumptions (e.g. Buckland et al. 1993; Marques 2004) unreliable (Alldredge et al. 2007; Efford and Dawson 2009). Furthermore, many of the statistical assumptions of distance sampling may be violated during field surveys, reducing accuracy of density estimates (Alldredge et al. 2007; Johnson 2008). For example, an important assumption of distance sampling is that distances to individual birds (an element of localization) are estimated accurately (Buckland et al. 1993); however, this is difficult to accomplish under normal field conditions (Alldredge et al. 2007; Johnson 2008; Efford and Dawson 2009) and violating this assumption leads to large errors in estimating avian densities (Marques 2004; Alldredge et al. 2007). While observers' ability to estimate distance to birds detected aurally in a forested ecosystem has been quantified (Alldredge et al. 2007), we are unaware of any studies that have determined accuracy of distance estimations in grassland ecosystems, or of effects of ambient noise on accuracy of distance estimates.

One way to quantify effects of anthropogenic noise on detectability and distance estimation during aural surveys is to use known locations of speakers as substitutes for real birds (Alldredge et al. 2007). We note that due to several differences between recordings played from speakers and real birds (e.g. absence of visual cues, movements of real birds during surveys, speakers were hidden by vegetation whereas territorial displays may be aerial), our methods do not allow us to quantify detectability of real grassland songbirds during normal point‐count and transect surveys. However, they are useful for allowing us to understand relative detectability in noisy and quiet areas, and to evaluate effects of masking and distraction on detectability and distance estimation error. We broadcast recordings of bird songs and pure tones at two separate frequencies (500 or 5300 Hz) in prairie sites with and without noise‐producing oil and gas extraction infrastructure while experienced observers used standard transect survey protocols to attempt to locate and identify the recordings. Similar studies were also conducted using point‐count surveys; as results were similar to results for transects, for conciseness we provide information on point‐count survey methods and results in Supporting Information only. We played tones to determine observers' general ability to detect standardized, unfamiliar stimuli in noisy and quiet environments, whereas avian songs were played to assess observers' ability to detect familiar avian species. We predicted greater effects of noise on detectability closer to infrastructure and for quieter bird songs. We hypothesized that if sound complexity improved detectability by reducing risk of masking all frequencies of the stimuli, songs would be more detectable than tones.

## Methods

### Study area

The study took place in native mixed‐grass prairie sites in south‐central Alberta, in landscapes with and without energy development, within 200 km of Brooks, Alberta, Canada (approximately 50° 53′ 58.091″ N, 112° 26′ 35.456″ W). Vegetation included grasses such as blue grama grass *Bouteloua gracilis* Willd. ex Kunth, northern wheatgrass *Elymus lanceolatus* (Scribn. & J.G. Sm.) Gould, and western wheatgrass *Pascopyrum smithii* (Rydb.) Á. Löve, forbs like pasture sage *Artemisia frigida* Willd., and a low abundance of silver sagebrush *Artemesia cana* Pursh and other shrubs. The height of vegetation in this region is on average 23.7 cm (SD = 19.4 cm, *n *=* *7504).

**Figure 1:**
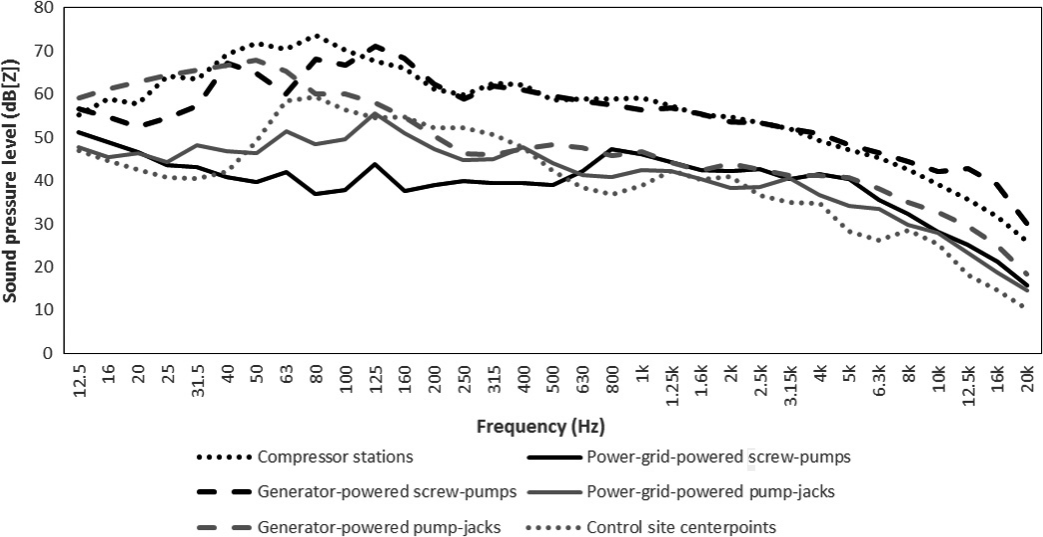
Sound pressure levels of ambient noise recorded at pump‐jacks, screw‐pumps, compressors, and control sites. Control site = 64‐ha section without oil or gas development within the study area. Time‐averaged SPLs of 1/3‐octave frequency bands were measured at 10 m from infrastructure and are expressed in dB(Z), or unweighted SPL. We excluded raw data and measures of variation to make all figures easier to read; measures of variation are shown in the text and tables.

We selected sites to represent a broad range of different types of natural gas and oil infrastructure that produced noise from low to high amplitudes and low to high frequencies (Fig. 1). We conducted transect surveys at 10 sites in 2013 (2 compressors, 3 generator‐powered pumpjacks, 2 power‐grid powered and 1 generator‐powered screw pump, and 2 controls), and 9 sites in 2014 (2 compressors, 2 generator‐powered pumpjacks, 1 power‐grid powered and 1 generator‐powered screw pump, and 3 controls; 2 of these sites were also surveyed in 2013). The point‐count study was conducted in 2012 (Supporting Information). This infrastructure was owned by Cenovus Energy, but is typical of infrastructure found industry‐wide in this region.

To characterize noise at our infrastructure sites, we measured time‐averaged sound pressure levels at 10 m from infrastructure using a Brüel and Kjær 2250 SPL meter/frequency analyzer (Brüel and Kjær, Nærum, Denmark). Gas compressor stations are structures that use turbines, motors, or engines to generate pressure to pump natural gas from wells through pipelines to central collection facilities, and produce relatively loud noise (82 dB(C) at 10 m; SD = 3.1, *n*_compressors_ = 4, one measurement in each cardinal direction unless obstruction prevented sampling: *n*_c‐total_ = 16 samples). Pumpjacks are aboveground structures that convert rotary motion from a motor to vertical motion in a piston pump at an oil well, drawing oil up from underground, and produce noise at average amplitudes of 68 dB(C) at 10 m (SD = 7.0, *n*_pumpjacks_ = 8, *n*_p‐total_ = 30). Screw pumps are positive displacement pump structures that use one or more rotating screws to generate pressure and draw viscous oil from the ground into pipes through cavities in the screws, and produce noise at similar amplitudes to pumpjacks (69 dB(C) at 10 m; SD = 10.7, *n*_screwpumps_ = 10, *n*_s‐total_ = 34). Both pumpjacks and screw pumps may be powered using generators, or may be powered by connecting infrastructure to the provincial power grid; generator‐powered wells produce noise at higher amplitude, particularly at low frequencies (Fig. 1). Cumulatively, average amplitude at 10 m from the infrastructure was 71 dB(C) (SD = 9.9, *n*_infrastructuresites_ = 22, *n*_total_ = 80). All these types of infrastructure produce noises at a range of low to high frequencies, with slightly more energy concentrated in the lower frequencies (Fig. 1). Control sites consisted of square 64‐ha sections of pasture with no oil wells and no gas compressors within 1000 m of a section's geographic center, within which no other anthropogenic noise sources were audible. While power of low‐frequency ambient noise in control sites was similar to power of low‐frequency ambient noise of grid‐powered well sites, it was lower than power of low‐frequency noise at sites with generators (Fig. 1). At frequencies that birds are most sensitive to, 1–6 kHz (e.g. Pater et al. 2009), ambient noise was lower at control than treatment sites (Fig. 1). When calculated across frequencies, ambient noise at control sites was on average 52 dB(C) at the centre (SD = 5.0, *n*_controls_ = 11).

### Playback stimuli

We created avian playback stimuli from Macaulay Library recordings (16‐bit samples, 44.1 kHz sampling rate, WAV file format) (Macaulay Library 2012). We chose 6–7 song examples from seven prairie bird species common to the study area to represent songs and calls that vary in characteristics that affect transmission (e.g. complexity, frequency range, and tonality): Marbled Godwit *Limosa fedoa* Linnaeus; Sprague's Pipit *Anthus spragueii* Audubon; Chestnut‐collared Longspur *Calcarius ornatus* Townsend; Vesper Sparrow *Pooecetes gramineus* Gmelin; Savannah Sparrow *Passerculus sandwichensis* Gmelin; Grasshopper Sparrow *Ammodramus savannarum* Gmelin; and Baird's Sparrow *Ammodramus bairdii* Audubon. We selected song samples with high signal‐to‐noise ratios and little to no conspecific or heterospecific interference in the recordings. We used a filter selection function to remove background noise (e.g. cars, human voices, and other birds) from our recordings between vocalizations, and a high‐pass and a low‐pass filter to remove background noise >2 kHz above or below, respectively, the highest and lowest frequencies of the vocalizations for each recording (Raven Pro 2012, Cornell Lab of Ornithology, Ithaca, NY). This procedure removed most of the background noise without clipping any of the focal bird's vocalizations, and removed vocalizations from nonfocal birds. We maintained the vocalization rate naturally occurring in each recording, and looped recordings to play continuously during point counts and transects.

To ensure that we played recorded songs at comparable amplitudes to those of real prairie birds, where possible, we calibrated the amplitude of the vocalizations by playing the recordings back to real birds (Baird's Sparrow, Chestnut‐collared Longspur, Horned Lark, Savannah Sparrow), while we observed from 20 m away. Once the real bird had approached the speaker to within 1 m and sung in response to our recording, we adjusted the iPod's amplitude to best match the real bird's amplitude. Once the iPod was calibrated, we measured the speaker's amplitude from 1 m away, using a Sound Pressure Level Meter (Pyle PSPL25; Pyle Audio Inc., Brooklyn, NY, USA) in C‐weighting, with slow response. It is difficult to measure the sound amplitude of our other focal species (Grasshopper Sparrow, Marbled Godwit, and Vesper Sparrow) because of their natural singing behaviors (e.g. singing in flight), and thus we categorized our focal species into “loud” and “quiet” song groups. To do this, we asked six experienced ornithology field technicians (not those subsequently tested) to classify our focal species into loud and quiet taxa groups. We then set mean amplitude of playback to 88 dB(C) at 1 m for “loud” birds (Baird's Sparrow, Chestnut‐collared Longspur, Marbled Godwit, Sprague's Pipit, Vesper Sparrow) and 84 dB(C) at 1 m for “quiet” birds (Grasshopper Sparrow, Savannah Sparrow), consistent with the amplitudes we recorded in the field.

In addition to the avian recordings, we used tones to examine the general ability of field technicians to detect and localize unfamiliar low and mid‐frequency sounds. We did not use higher frequencies because previous studies have demonstrated that high‐frequency sounds do not propagate very far due to their physical properties (Cosens and Falls 1984; Romer and Lewald 1992), and are difficult to localize (Bronkhorst 1995), and thus we felt it was unnecessary to repeat these studies. We created 5‐min, pure‐tone stimuli at 500 and 5300 Hz with sine‐wave‐generating software (Test Tone Generator; Esser Audio, Greensburg, PA). These frequencies represent the lowest frequency and average of the median frequencies found in all of our species' vocalizations.

### Experimental procedures

We installed speakers (PureAcoustics HipBox Portable Audio Speakers) at predetermined randomized distances (5–100 m) from transect lines, and 10–400 m along transect lines radiating away from infrastructure (or center of the control sites). We did not use song exemplars more than once at a site. We used a stratified random approach to selecting speaker distances from the center line to ensure that we had similar numbers of nearby and distant speakers at noisy and control sites. We used iPod Nanos to play songs or tones (different players for each experiment). Different technicians set up sites relative to those who conducted surveys. We varied numbers of speakers per transect so that observers could not predict the number of recordings.

Prior to starting the experiment, we calibrated speakers to either each species' approximate natural amplitude (i.e., 88 or 84 dB[C]), or, for tones, the speaker's maximum amplitude (97 dB[C]). Calibration for song amplitude was accomplished using a Pyle SPL meter, at 1 m from the speaker.

We conducted the transect experiment using 3–5 experienced observers per site in 2013 and nine experienced observers per site in 2014, at two noise levels: noisy infrastructure sites, e.g. sites with active generator‐powered pumpjacks, compressor stations, or screw pumps (*n* = 81 surveys for songs and tones, each); and quiet sites, i.e. control sites without infrastructure (*n* = 35 surveys for songs and tones, each). Preliminary analyses suggested no detectable difference in effects of noise from different types of infrastructure, so infrastructure types were collapsed into a binary control/infrastructure independent variable. Although this approach added within‐treatment variability, it ensured our “noisy” treatment reflected the frequencies and amplitudes of sounds produced by a wide range of different types of anthropogenic infrastructure (Fig. 1). We conducted 400‐m, 40‐min long transects (one per observer per site). Transects either started as close as possible to infrastructure (following health and safety guidelines, usually approximately 7 m from infrastructure), or at the control site center point; or at the opposite end of the transect.

Surveys were conducted 1–6 August in 2013 and 21–30 July in 2014, when there was no rain and wind <15 km/h. We used a single transect per observer at each site for both songs and tones, looping recordings to repeat continuously. We used tone recordings in which a tone played for 7 sec, then was quiet for 30 sec before the recording was repeated. In 2014, to ensure that we had a minimum sample size of available observations for each species, we changed recording selection to ensure songs of each species were projected at each site.

During each transect survey, each observer determined the distance (m), direction (degrees), and species or type of each song or tone that they heard, consistent with the methods we use for transect surveys of real birds. Each time the observer noted a bird song or tone, they recorded the observer's distance from the start of the transect using a GPS unit (Garmin GPS 60 ± 2–5 m accuracy).

### Analyses

We used generalized linear mixed models (lmer and glmer functions in R [Bates et al. 2014]) to evaluate effects of noise and distance to recordings and infrastructure on detectability and localization accuracy. We compared analyses using site or observer as a random effect to account for potential correlations in data due to repeated measurements per site and per observer (Bolker et al. 2008). Response variables were whether or not a song or tone was detected (binary variable), and distance error (absolute value of (estimated distance to observer – actual distance to observer); this response variable followed a normal distribution).

We used a frequentist approach (Mundry 2011) for statistical analyses (*α *= 0.05). We first evaluated whether a quadratic term (in addition to the linear term) for speaker distance to transect line (DT: transects) was required, to allow for this relationship to be nonlinear. If the quadratic term was not significant in the preliminary model, we removed it from the model to reduce the likelihood of collinearity and increase parsimony (Quinn and Keough 2002). We then added variables to the model, including frequency for tones (P: high or low) and species for songs (SPP: Baird's Sparrow, Chestnut‐collared Longspur, Grasshopper Sparrow, Marbled Godwit, Savannah Sparrow, Sprague's Pipit, and Vesper Sparrow; detectability of each species was compared against the species (reference level) that our empirical results demonstrated had the highest detectability [Baird's Sparrow]); ambient noise level (NL: infrastructure site/control site); and speaker distance from infrastructure (DI). If distance or ambient noise level main effects were significant, we then tested for significant interactions between speaker distance from infrastructure and ambient noise level, and speaker distance from the transect line and ambient noise level. If interaction terms were statistically significant, the model including the interaction term was the final model. If interaction terms were not statistically significant, the model containing only main effects became the final model, to minimize problems with collinearity caused by interaction terms, and increase parsimony (Quinn and Keough 2002). Thus, base models with interactions but without quadratic terms were as follows:

Tones: Response variable = DT + P + NL + DT*NL + DI + DI*NL
Songs: Response variable = DT + SPP + NL + DT*NL + DI + DI*NL

## Results

Including different random effects in models did not alter our conclusions. Site accounted for more model variance than observer, so to be concise, we present only the results from the models with site as the random effect.

**Figure 2:**
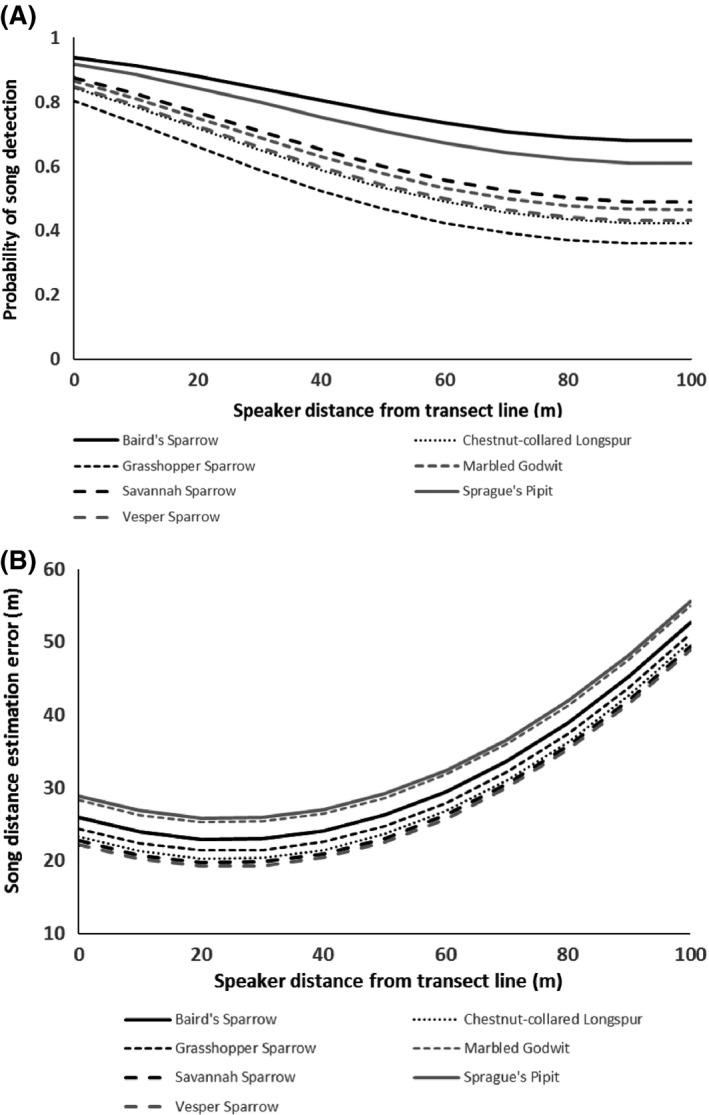
(A) Probability of song detection and (B) song distance estimation error in southern Alberta grasslands, 2013–2014. Both variables were affected by speaker distance from the transect line, but only detection probability varied with species.

Probability of detecting songs decreased with increasing speaker distance from the transect line, and varied among species (Fig. 2A, Table 1). Songs of all species except for Sprague's Pipits were significantly less likely to be detected than Baird's Sparrows, with Grasshopper Sparrow songs having the lowest probability of detection. Effects of year, distance from infrastructure, ambient noise level, and interactions were insignificant in all models, indicating that ambient noise had no effect on detectability (Table 1). Error in estimating song speaker distance from the observer increased as a quadratic function of increasing speaker distance from the transect line, but was not influenced by species, ambient noise, or any other variables or interactions (Fig. 2B, Table 1).

**Table 1:** Parameter effect sizes ± standard errors (*P*‐values) for probability of detecting song recordings and for error in estimating distance to song recording (¦actual − estimated speaker distance from observer¦ [m]) of songs during transects in southern Alberta prairies, 2013–2014. Detectability of each species was compared with the species with highest detectability, Baird's Sparrow. Interaction terms DT*NL and DI*NL were not included in any final model because they were never significant. Tone distance estimation error, but not detection probability, varied as a quadratic function of speaker distance from transect

	Probabilility of detection	Error in distance estimation (m)
Intercept	3.109 ± 0.605 (<0.001)	33.392 ± 8.472 (<0.001)
Year	−0.251 ± 0.241 (0.208)	−3.816 ± 3.710 (0.247)
Speaker distance from transect (DT [m])	−0.042 ± 0.011 (<0.001)	−0.254 ± 0.216 (0.208)
Speaker distance from transect^2^	0.0002 ± 0.0001 (0.024)	0.005 ± 0.002 (0.007)
Species		
Chestnut‐collared Longspur	−1.065 ± 0.297 (<0.001)	−2.633 ± 4.270 (0.539)
Grasshopper Sparrow	−1.331 ± 0.326 (<0.001)	−1.540 ± 5.088 (0.797)
Marbled Godwit	−0.892 ± 0.348 (0.010)	2.372 ± 4.944 (0.668)
Savannah Sparrow	−0.796 ± 0.303 (0.009)	−3.173 ± 4.260 (0.429)
Sprague's Pipit	−0.305 ± 0.304 (0.316)	2.935 ± 4.025 (0.463)
Vesper Sparrow	−1.030 ± 0.299 (<0.001)	−3.684 ± 4.671 (0.394)
Ambient noise level (NL [quiet = 1])	0.136 ± 0.281 (0.630)	−5.269 ± 4.354 (0.188)
Distance from infrastructure (DI [m])	0.0002 ± 0.001 (0.836)	0.003 ± 0.012 (0.857)

**Figure 3:**
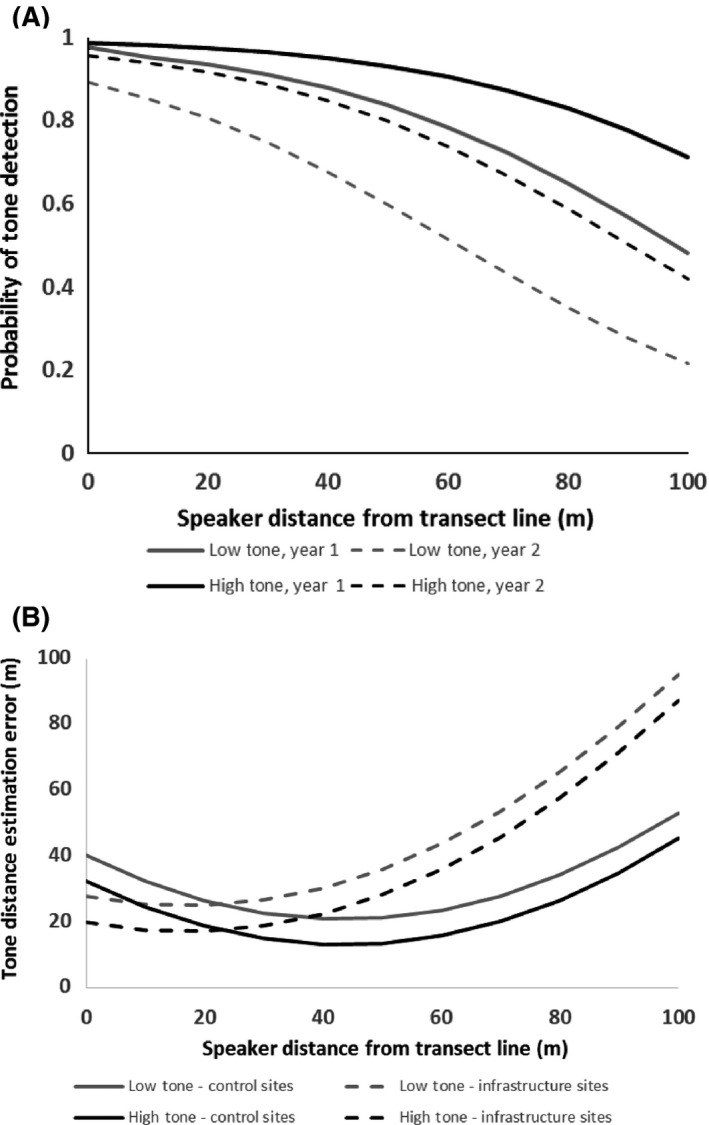
(A) Probability of tone detection and (B) tone distance estimation error in southern Alberta grasslands, 2013–2014. Both variables were affected by speaker distance from the transect line. Tone detection probability also varied with year and tone frequency, while tone distance estimation error depended on whether transect was at a control or infrastructure site.

Probability of detecting tones was relatively consistent within 50 m of the transect line, but declined at greater distances (Fig. 3A). Probability of detecting tones was lower in the second year of surveys and for low‐frequency tones, but was not influenced by ambient noise or other variables or interactions (Fig. 3A, Table 2). Errors in estimating tone distance from the transect line increased as a quadratic function of increasing speaker distance from the transect line, at steeper rates at infrastructure sites than control sites (Fig. 3B, Table 2).

**Table 2:** Parameter effect sizes ± standard errors (*P*‐values) for probability of detecting tones and for error in estimating distance from observer to tone (¦actual − estimated speaker distance from observer¦ [m]) during transects in southern Alberta prairies, 2013–2014. Tone distance estimation error, but not detection probability, varied as a quadratic function of speaker distance from transect

	Probability of detection	Error in distance estimation (m)
Intercept	5.676 ± 1.312 (<0.001)	30.934 ± 15.363 (0.033)
Year	−1.228 ± 0.495 (0.013)	−8.848 ± 7.346 (0.182)
Speaker distance from transect (DT [m])	−0.034 ± 0.009 (<0.001)	−0.342 ± 0.414 (0.412)
Speaker distance from transect^2^		0.010 ± 0.004 (0.012)
Frequency (Low = 1)	−0.973 ± 0.420 (0.020)	7.820 ± 5.893 (0.208)
Ambient noise level (NL [quiet = 1])	0.269 ± 0.541 (0.618)	9.904 ± 21.469 (0.535)
DT*NL		−0.544 ± 0.226 (0.012)^a^
Distance from infrastructure	0.001 ± 0.002 (0.596)	0.013 ± 0.029 (0.575)
DI*NL		0.012 ± 0.070 (0.962)

a The significant interaction term between noise level and speaker distance from the transect line indicates that error in distance estimation increased with increasing speaker distance from observers at greater rates at loud or infrastructure sites than at quiet or control sites.

## Discussion

We did not find strong effects of noise on detectability or localization accuracy. These results differed from other studies that have evaluated effects of compressor station noise on detectability (Blickley and Patricelli 2010; Ortega and Francis 2012), but are somewhat consistent with the observations of Pacifici et al. (2008), who found that detections within 50 m of observers were independent of ambient noise levels. Surprisingly, given that infrastructure transects started or ended immediately adjacent to noisy infrastructure, there was no effect of ambient noise on detectability. Noise may act as a distraction from the execution of relatively complex cognitive tasks (Beaman 2005) but it is much less likely to interfere with the completion of simple cognitive tasks (Howard et al. 2010). For experienced observers, the task of conducting avian surveys may not be sufficiently complex to be susceptible to distraction by background noise.

The only significant effect of ambient noise was that distance estimation errors for tones were higher in noisy sites, and errors increased as distance from the transect line increased. The signal‐to‐noise ratio decreases with increased distance from the signal (Brumm and Slabbekoorn 2005), and since signal detection is heavily dependent on this ratio (Brumm and Slabbekoorn 2005), when observers are further away from the signal source, their ability to accurately localize a cue is reduced. However, this effect was not observed for songs. This may be because songs are complex sounds composed of numerous frequencies, with low frequencies normally travelling greater distances compared to high frequencies (International Organization for Standardization 1996). Consistent with our predictions, the complexity of bird songs may ensure that some frequencies within the song reach the observer, even if higher frequencies do not. Furthermore, because songs contain many frequencies, this may reduce the likelihood of ambient noise masking the entire song. In contrast, pure tones consisted of one frequency, which was overlapped by the broad range of background noise frequencies produced by oil and gas infrastructure, perhaps making tones more susceptible to masking (Dooling and Blumenrath 2013). Alternatively, we speculate that it might be easier to distinguish from background noise familiar songs to which observers have been repeatedly exposed, compared with unfamiliar tones.

We found little evidence that ambient noise distracted observers from their tasks. This may be because anthropogenic noise at our sites was produced at a single point source, and was repetitive and predictable. Poor performance of a task is more likely to occur when distracting noise are characterized by sudden frequency changes and diverse rhythms (Beaman 2005). This suggests that during avian surveys, observers might be less susceptible to distraction by consistent, predictable ambient noises (e.g. energy infrastructure) than to unpredictable or variable ambient noises (e.g. traffic, airplanes, construction noise).

As in some previous studies (Kendall et al. 1996; Diefenbach et al. 2003; Moore et al. 2004), songs of different species varied in detectability. However, our prediction that quiet songs would be less detectable than loud songs was not supported, as detectability of Savannah Sparrows, a “quiet” species, was not lower than detectability of other species. We speculate that relatively low detectability of Grasshopper Sparrow songs were because they were both quiet and high‐frequency (see also Diefenbach et al. 2003); a large portion of their song (ca. 4–11 kHz; Vickery 1996) is above the peak human hearing sensitivity of 1–6 kHz (Davis 2007). Detectability of low tones was also lower than for mid‐frequency tones. Songs of larger birds, which tend to have lower average frequencies (e.g., Francis et al. 2011), may be less detectable than songs of smaller birds; however, in grasslands, this effect may be compensated for by the higher visibility of larger birds.

Songs and tones showed qualitatively similar trends, in that detectability was generally higher near observers, and distance estimation errors increased as distances to observers increased. However, detectability and localization accuracy were generally higher for tones than songs, in contrast with our predictions. We speculate that the high distance estimation errors we found for songs were in part because experienced observers expected to use additional cues, such as visual identification, to localize songs (inferential approach to interpreting stimuli; Lutfi 2008; King 2009); their absence may have confused observers and increased errors. Accurate detection of acoustic cues tends to increase in the presence of associated visual cues (King 2009). Because playback experimental designs provide an incomplete set of signals (acoustic only) to observers, associated information loss could incite observers to infer from prior knowledge that an individual bird heard was not actually present (Lutfi 2008). Furthermore, observers who participated in the experiment reported that it was difficult to determine the location of songs that would normally be performed aerially, such as Sprague's pipits (Robbins 1998). Thus, prior natural history knowledge could have hindered detections of birds' acoustic cues during our artificial surveys. Distance estimation errors of tones, for which observers had no prior knowledge, may more accurately reflect the ability of observers to estimate locations of sounds.

This result highlights the fact that our playback experiment cannot perfectly replicate the conditions of field surveys. For example, we provided only stationary acoustic cues, but many grassland birds are seen or heard singing or calling while in flight. The addition of visual cues during real bird surveys could increase detectability; conversely, avian movement during surveys could increase distance estimation error. Nonetheless, we suggest that our design was reasonable for evaluating whether detection and localization of acoustic cues using typical avian survey methods are impeded by ambient noise, and whether localization ability declines with distance to observer.

Although many studies have discussed imperfect detectability of songbirds during aural surveys (e.g., Anderson 2001; Efford and Dawson 2009), few have quantified the ability of observers to estimate locations of birds (Marques 2004). In a densely forested ecosystem, Alldredge et al. (2007) demonstrated large errors in distance estimates, which varied unpredictably as distance between speaker and observer increased. Similarly, we found that in prairies, distance estimation errors were large. However, in our ecosystem, errors generally increased predictably as distance to the observer increased. While distance estimation errors can be compensated for in some ecosystems by using rangefinders to estimate distances to likely perch sites (Alldredge et al. 2007; Greene et al. 2010), this is not effective in grasslands, where some individuals sing from below the vegetation canopy, or aerially, and habitat structure usually cannot be used to predict their locations. While training results in small increases in accuracy, errors are still likely (Alldredge et al. 2007).

These trends, in decreasing detectability and increasing distance estimation errors as distances to birds increase, present a dilemma for surveyors. It is generally assumed that decreasing detectability with increasing distance can be compensated for by using distance sampling (e.g. Buckland et al. 1993). However, inaccurate distance estimates violate assumptions of distance sampling (Buckland et al. 1993), and result in large biases in estimating densities (e.g., Alldredge et al. 2007). Methods that have been developed to compensate for imperfect detectability and localization (Marques 2004) increase variance and decrease precision as a result of their added complexity (Efford and Dawson 2009), and include additional assumptions regarding distribution of the error function that may not be met (Alldredge et al. 2007). Thus, we cannot recommend using distance sampling for species whose distances to observers cannot be confirmed using visual estimates (see also Alldredge et al. 2007). Unfortunately, other methods for compensating for imperfect detectability are also problematic in grassland ecosystems (Leston et al. 2015). Assumptions of population closure for removal sampling are violated when birds move during surveys (Farnsworth et al. 2002), a common occurrence with grassland species (Roberts and Schnell 2006; Davis et al. 2013; Kalyn Bogard and Davis 2014). Reducing survey extents covered by point‐counts or transects (e.g., to 50 m instead of 100 m radii) would reduce but not eliminate imperfect detectability, increase the likelihood of violating the assumption of population closure, and result in lower statistical power because sample size declines if survey areas are smaller (Quinn and Keough 2002). Similarly, double‐ or multiple‐observer sampling (Nichols et al. 2000; Alldredge et al. 2006) reduces but does not eliminate problems with detecting distant individuals, and reduces power by decreasing the area that can be sampled given fixed resources (Leston et al. 2015). This suggests that no available statistical method or study design offers a particularly satisfactory solution to the problem of imperfect detectability of unseen birds at this time (see also Efford and Dawson 2009; Amundson et al. 2014).

Nonetheless, our results provide us with some confidence that many of the previous studies that have used field surveys to evaluate effects of noisy anthropogenic features on birds (e.g., Ingelfinger and Anderson 2004; Summers et al. 2011) have probably been robust, in that results may not have been driven by effects of noise on detectability. However, this risk must be considered when designing such studies (e.g., Francis et al. 2009).

## Conflict of Interest

None declared.

## Supporting information


**Data S1.** Methods.
**Table S1**. Parameter effect sizes ± standard errors (*P*‐values) for probability of detecting song recordings (*n* = 110 recordings played to all observers) and for error in estimating distance (¦actual‐estimated speaker distance from observer¦ [m]) of songs (*n* = 69 recordings detected by any observer) to observers during point counts in 2012.
**Table S2.** Parameter effect sizes ± standard errors (*P*‐values) for probability of detecting tones (*n* = 125 recordings played to all observers) and for error in estimating tone distance (¦actual – estimated speaker distance from observer¦ [m]) of songs (*n* = 81 recordings detected by any observer) during point counts in 2012.
**Figure S1.** (a) Probability of song detection and (b) distance estimation error in 2012.
**Figure S2.** (a) Probability of tone detection and b) tone distance estimation error in 2012.
