# Supplementary material for: Effects of ambient noise on detectability and localization of avian songs and tones by observers in grasslands
Source: Ecol Evol. 2015 Dec 29;6(1):245–55. doi: 10.1002/ece3.1847 (PMC4716498; doi:10.1002/ece3.1847)
Supplement: Supplementary file 1 — Data S1. Methods. Table S1. Parameter effect sizes ± standard errors (P‐values) for probability of detecting song recordings (n = 110 recordings played to all observers) and for error in estimating distance (¦actual‐estimated speaker distance from observer¦ [m]) of songs (n = 69 recordings detected by any observer) to observers during point counts in 2012. Table S2. Parameter effect sizes ± standard errors (P‐values) for probability of detecting tones (n = 125 recordings played to all observers) and for error in estimating tone distance (¦actual – estimated speaker distance from observer¦ [m]) of songs (n = 81 recordings detected by any observer) during point counts in 2012. Figure S1. (a) Probability of song detection and (b) distance estimation error in 2012. Figure S2. (a) Probability of tone detection and b) tone distance estimation error in 2012. [file ECE3-6-245-s001.docx]

SUPPORTING INFORMATION

*Point count experiment*

METHODS

In addition to the transect study described in the body of this paper, we conducted a similar study on detectability of bird songs and tones at 5-minute, 100-m fixed-radius point-count plots within 7 sites in 2012 (2 natural gas compressor stations, 1 generator-powered pumpjack, 1 power-grid powered screw pump, and 3 controls with no anthropogenic infrastructure).

This experiment was to evaluate effects of low-amplitude noise on detectability of songs and tones during point-count plot surveys. We conducted the point-count experiment with 5 experienced observers, at 2 plots in each site. The order of observers and sites was randomized to control for order effects. Each observer conducted one 5-minute survey for songs followed by one 5-minute survey for tones within the same plot. The ability of technicians to accurately detect and locate sounds during point-count surveys were tested at two noise levels: noisy infrastructure sites, e.g. sites with active generator-powered pumpjacks, compressor stations, or screw pumps (*n* = 40 surveys for songs and tones, each); and quiet sites, i.e. control sites without infrastructure (*n* = 30 surveys for songs and tones, each). Preliminary analyses suggested no detectable difference in effects of noise from different types of infrastructure, so infrastructure types were collapsed into a binary control/infrastructure independent variable.

Point count plot centers and point count observers were approximately 300-350 m from the infrastructure and control site center-points. Amplitude at this distance was low (49-56 dB[C], SD = 4.0-8.0) but infrastructure was audible.

We set up point counts over four mornings (1-6 August, 2012, between dawn and 10:00h, without rain, winds < 15 km/h), after the avian breeding season in this area. This was to ensure there were few singing birds in the survey sites; we abandoned an earlier attempt at the study in July, when playbacks solicited responses and visits from many wild birds, artificially increasing detectability of the playbacks.

Setup for transects was similar to that of point counts in terms of dates, equipment, weather (no rain, wind < 15 km/h), song species and tones used in recordings, and variation in the numbers, locations, types of recordings per transect, and placement of speakers at random distances from the center point of the plot (5-100 m). Setup of speakers and WAV players differed in that we attached WAV players to the speakers via long cables, which allowed us to turn players on and off from a central location.

Statistical analyses were the same as for transect analyses, except excluding distance to infrastructure as an independent variable. The reference category against which detectability of other species was compared was Sprague’s pipit, as it had the highest detectability during point-count surveys. For probability of song detection, we repeated analyses without Chestnut-collared Longspur recordings, because detectability of this species was 1.0, resulting in 0 variability within that species treatment, which prevented model convergence.

RESULTS

Probability of detecting songs declined with increasing speaker distance from point-count observers, and varied among species but not between infrastructure (loud) and control (quiet) sites (Figure Sup1A, Table Sup1). Baird’s Sparrow, Grasshopper Sparrow, Marbled Godwit, and Savannah Sparrow songs were less likely to be detected than Sprague’s Pipit songs (Figure Sup1A, Table Sup1). Error in estimating distance to recording was lower for Baird’s Sparrows, Chestnut-collared Longspurs, Grasshopper Sparrows, Savannah Sparrows, and Vesper Sparrows than Sprague’s Pipits, but did not vary significantly with speaker distance from observer or ambient noise level (Figure Sup1B, Table Sup1).

Probability of detecting tones declined with increasing speaker distance from observer, but did not vary significantly with frequency or ambient noise level (Figure Sup1A, Table Sup2). Error in estimating distances of tones to the observer increased with increasing speaker distance from observers, but did not vary significantly with frequency or ambient noise level (Figure Sup2B, Table Sup2).

TABLE Sup1. Parameter effect sizes + standard errors (p-values) for probability of detecting song recordings (n = 110 recordings played to all observers) and for error in estimating distance (|actual-estimated speaker distance from observer| [m]) of songs (n = 69 recordings detected by any observer) to observers during point counts in 2012. Detectability of each species was compared with detectability of Sprague’s Pipits. Note: †Chestnut-collared Longspurs were not included in the probability analysis because all longspur recordings were detected by all observers. The interaction term DO*NL and the quadratic term for speaker distance from observer were not included in any model because they were never significant.

|  | Probability of detection | Error in distance estimation (m) |
| --- | --- | --- |
| Intercept | 5.112 ± 1.315 (0.001) | 44.353 ± 9.942 (<0.001) |
| Speaker distance from observer (DO [m]) | -0.044 ± 0.011 (<0.001) | -0.026 ± 0.128 (0.396) |
| Species: |  |  |
| Baird’s Sparrow | -2.187 ± 0.970 (0.024) | -10.619 ± 8.092 (0.070) |
| Chestnut-collared Longspur | † | -24.082 ± 11.008 (0.024) |
| Grasshopper Sparrow | -4.164 ± 1.282 (0.001) | -21.298 ± 12.040 (0.030) |
| Marbled Godwit | -5.180 ± 1.499 (<0.001) | 11.436 ± 21.024 (0.961) |
| Savannah Sparrow | -2.426 ± 1.049 (0.021) | -17.671 ± 7.531 (0.010) |
| Vesper Sparrow | -0.595 ± 1.163 (0.609) | -23.780 ± 11.061 (0.012) |
| Ambient noise level (NL [quiet=1]) | -0.506 ± 0.607 (0.405) | -4.817 ± 9.270 (0.322) |

TABLE Sup2. Parameter effect sizes + standard errors (p-values) for probability of detecting tones (n = 125 recordings played to all observers) and for error in estimating tone distance (|actual - estimated speaker distance from observer| [m]) of songs (n = 81 recordings detected by any observer) during point counts in 2012. The interaction term DO*NL and the quadratic term for speaker distance from observer were not included in any model because they were never significant.

|  | Probability of detection | Error in distance estimation (m) |
| --- | --- | --- |
| Intercept | 2.717 ± 0.569 (<0.001) | 8.323 ± 5.403 (0.100) |
| Speaker distance from observer (DO [m]) | -0.048 ± 0.010 (<0.001) | 0.251 ± 0.083 (0.003) |
| Frequency (Low=1) | 0.202 ± 0.468 (0.667) | 4.493 ± 3.822 (0.230) |
| Ambient noise level (NL [quiet=1]) | 0.632 ± 0.456 (0.166) | -0.498 ± 5.370 (0.946) |


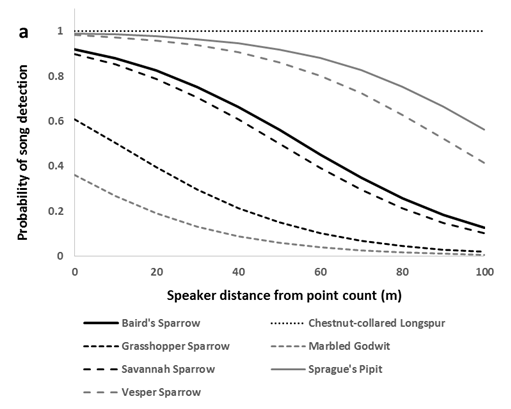

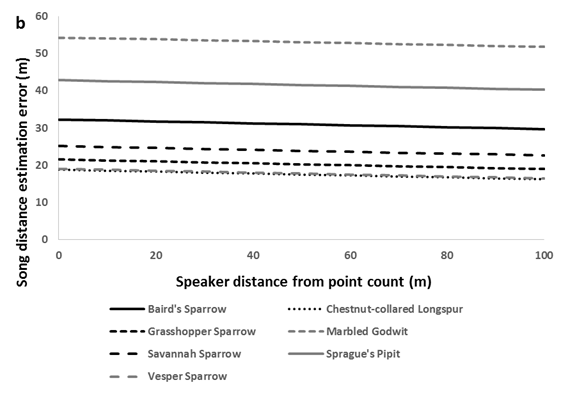


Figure Sup1. a) Probability of song detection and b) distance estimation error in 2012. Both variables differed by species, but only detection probability varied with speaker distance from the point count. For clarity, here and for subsequent figures, we show only predicted curves, as confidence intervals add too many additional lines to the figures for them to be interpretable.


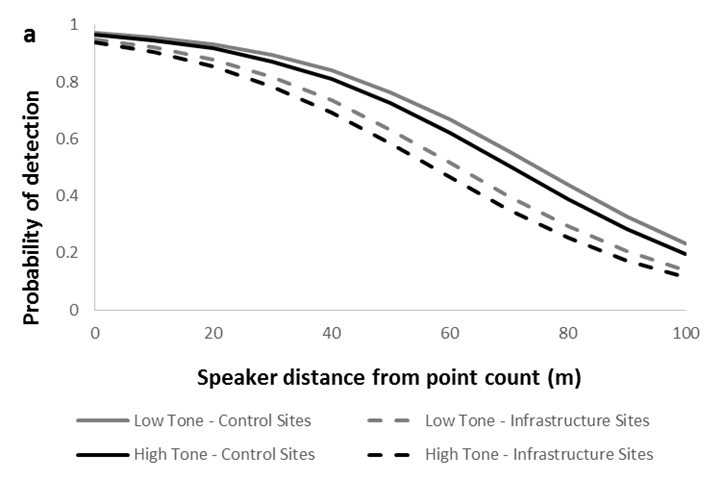

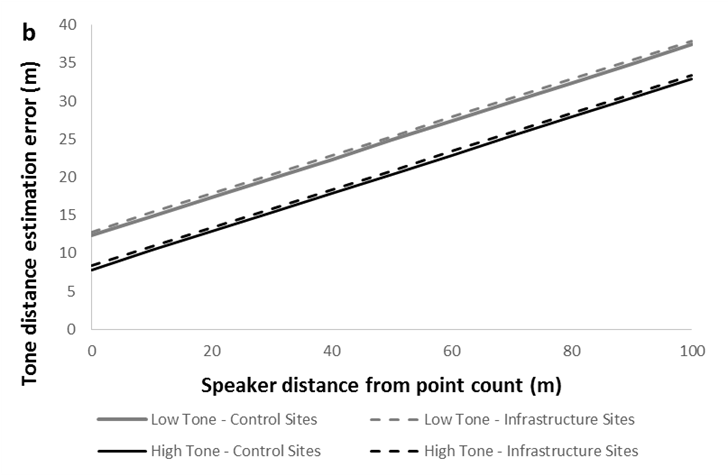


Figure Sup2. a) Probability of tone detection and b) tone distance estimation error in 2012. Both variables were affected by speaker distance from the point count, and distance estimation error also depended on whether point count was at a control or infrastructure site.
